# *Spartina anglica* extract attenuates TNF-α/IFN-γ-induced inflammatory responses in HaCaT keratinocytes through suppression of the MAPK, NF-κB, and STAT1 signaling pathways

**DOI:** 10.3389/fphar.2026.1931934

**Published:** 2026-10-30

**Authors:** Yuna Ha, Yewon Kim, Jeong-In Park, Jang Kyun Kim, Miles F. Wilkinson, Youn-Jung Kim

**Affiliations:** 1 Research Institute of Basic Sciences, Incheon National University, Incheon, Republic of Korea; 2 Department of Obstetrics, Gynecology, and Reproductive Sciences, University of California San Diego, La Jolla, CA, United States; 3 Department of Marine Sciences, Incheon National University, Incheon, Republic of Korea; 4 Institute of Genomics, University of California San Diego, La Jolla, CA, United States

**Keywords:** anti-inflammatory, halophyte, keratinocytes, MAPK signaling, NF-κB signaling, *Spartina anglica*

## Abstract

This study investigated the anti-inflammatory effects of *Spartina anglica* extract (SAE) in a human keratinocyte model of inflammation. HaCaT cells were stimulated with tumor necrosis factor-α (TNF-α) and interferon-γ (IFN-γ) to induce inflammatory responses. Cell viability was assessed using the MTT assay to determine non-cytotoxic concentrations. The expression of the chemokines TARC/CCL17 and macrophage-derived chemokine MDC/CCL22 was quantified at both the mRNA and protein levels using quantitative real-time PCR and ELISA, respectively. In addition, the activation of key inflammatory signaling pathways was evaluated by Western blot analysis. SAE did not exhibit cytotoxic effects at concentrations up to 250 μg/mL. Under inflammatory conditions, SAE significantly reduced the expression of CCL17 and CCL22 in a dose-dependent manner. Mechanistically, this suppression was associated with decreased phosphorylation of MAPK signaling components, including ERK, JNK, and p38, as well as inhibition of NF-κB and STAT1 activation. High-performance liquid chromatography analysis revealed a major peak corresponding to the retention time of iso-orientin in SAE. These findings suggest that SAE may attenuate keratinocyte-mediated inflammatory responses through modulation of chemokine expression and key intracellular signaling pathways.

## 1 Introduction

Coastal salt marshes are widely recognized for their role in maintaining environmental stability and supporting surrounding ecosystems. They help reduce the impact of waves and wind along coastlines, which in turn contributes to sediment stabilization. More recently, these ecosystems have also been highlighted as important blue carbon sinks, as they can capture atmospheric carbon dioxide and store organic carbon in sediments over long periods of time (Barbier et al., 2011; Chmura et al., 2003). Coastal salt marshes are typically dominated by halophytic vegetation adapted to saline environments, including species such as *Sporobolus anglicus* (C.E.Hubb.) P. M. Peterson and Saarela [Poaceae, syn.: *Spartina anglica* C.E.Hubb.]. This plant has been reported to contribute to ecosystem stability by mitigating coastal erosion and facilitating long-term carbon storage within sediment systems (Barbier et al., 2011; Gore et al., 2024).

*S. anglica* is recognized for its ability to tolerate highly saline environments and for its diverse repertoire of secondary metabolites (Halsall et al., 2008; Mishra and Tanna, 2017), features that have contributed to its ecological relevance. However, in certain coastal regions, including South Korea, this species has also been classified as invasive due to its association with disruption of native ecosystems and declines in biodiversity (Kim et al., 2023). Despite these ecological considerations, the bioactive metabolites and pharmacological properties of *S. anglica* have not yet been extensively characterized (Ksouri et al., 2012). Beyond its ecological classification, *S. anglica* represents a growing management burden in Korean coastal ecosystems, having required costly removal since first being reported on Ganghwa Island in 2012 (Kim et al., 2015) and generating a biological waste stream with its own disposal costs (Shin et al., 2024). Identifying pharmacologically active metabolites in this biomass therefore supports the valorization of invasive coastal biomass rather than its disposal as waste.

A previous study reported the antioxidant, tyrosinase-inhibitory, and pancreatic lipase-inhibitory activities of *S. anglica* and identified several putative bioactive metabolites (Kim et al., 2021). Although only a limited number of pharmacological studies have been conducted on *S. anglica*, available evidence suggests that it possesses antioxidant, tyrosinase-inhibitory, pancreatic lipase-inhibitory, and anti-inflammatory activities. However, its anti-inflammatory activity has not yet been investigated in keratinocyte-mediated skin inflammation, despite the growing interest in keratinocyte-targeted bioactive metabolites for cosmetic and pharmaceutical applications. Therefore, the present study aimed to evaluate the anti-inflammatory potential of SAE and elucidate the underlying molecular mechanisms in TNF-α/IFN-γ-stimulated HaCaT keratinocytes.

Natural products derived from marine plants and algae have gained increasing interest in biomedical and pharmaceutical research due to their diverse biological activities, including antioxidant, anti-inflammatory, and immunomodulatory effects (Cheung et al., 2016). In particular, secondary metabolites such as polyphenols, flavonoids, and steroids have been shown to influence disease-related processes by modulating pro-inflammatory signaling pathways (Pereira and Cotas, 2023). For instance, fucoxanthin isolated from brown algae has been reported to exert multiple pharmacological effects, including anti-obesity, antibacterial, and skin-related activities (Maeda et al., 2006). In addition, marine-derived polyphenolic metabolites, such as phlorotannins, have been shown to exhibit anti-inflammatory properties through the regulation of inflammatory mediators and attenuation of oxidative stress (Wijesinghe and Jeon, 2012; Kok et al., 2023; Kim A.-R. et al., 2024. Despite the extensive investigation of marine algae, particularly in East Asia, *S. anglica* has received comparatively limited attention as a potential source of bioactive metabolites (Giordano et al., 2021). Although this halophytic species has been well studied in terms of its ecological functions and adaptive responses to environmental stress (Bortolus et al., 2019), its pharmacological properties remain insufficiently characterized. In particular, it is still unclear whether *S. anglica* contains metabolites with anti-inflammatory activity. In this study, we examined the bioactive metabolites of *S. anglica* and evaluated their anti-inflammatory effects. Furthermore, we assessed its potential applicability as a natural resource for pharmacological use.

Impaired immune regulation and sustained inflammatory signaling are central features in the development and progression of inflammatory diseases, including those affecting the skin. Among these, atopic dermatitis (AD) is a representative chronic inflammatory disorder characterized by the combined effects of immune imbalance, epidermal barrier dysfunction, and persistent inflammation (Bieber, 2008). A key aspect of AD pathogenesis is the elevated expression of thymus and activation-regulated chemokine (TARC/CCL17) and macrophage-derived chemokine (MDC/CCL22), which contribute to disease progression by promoting the recruitment and activation of T helper 2 (Th2) cells and eosinophils (Horikawa et al., 2002). These chemokines are therefore considered reliable indicators of disease severity and inflammatory activity in AD (Jahnz-Rozyk et al., 2005). The MAPK, NF-κB, and STAT1 signaling pathways are well-established regulators of TNF-α/IFN-γ-induced inflammatory responses in keratinocytes and play critical roles in controlling the expression of inflammatory mediators, including CCL17 (TARC) and CCL22 (MDC) (Ha et al., 2022; Yano et al., 2015). MAPK signaling regulates inflammatory responses through phosphorylation cascades, whereas NF-κB and STAT1 function as key transcriptional regulators downstream of TNF-α and IFN-γ signaling, respectively. Together, these pathways coordinate the expression of numerous pro-inflammatory genes and therefore represent major molecular targets for investigating the anti-inflammatory mechanisms of candidate therapeutic agents. Accordingly, the present study examined whether *S. anglica* extract modulates these signaling pathways in TNF-α/IFN-γ-stimulated HaCaT keratinocytes.

Recent studies have highlighted that natural products derived from marine organisms possess a wide range of biological activities, including immunomodulatory and anti-inflammatory effects, suggesting their potential applicability in the treatment of inflammatory disorders such as skin diseases (Cheung et al., 2016). In this context, despite its ecological relevance and biochemical diversity, *S. anglica* has not been sufficiently investigated for its role in inflammatory regulation. To determine whether *S. anglica* extract can modulate inflammatory responses in keratinocytes, an *in vitro* model based on TNF-α/IFN-γ-stimulated HaCaT cells was employed (Kong et al., 2015). In addition, changes in the expression of key inflammatory biomarkers associated with AD were examined to better understand its potential mechanisms of action.

To further characterize these effects, the phytochemical composition of *S. anglica* extract was analyzed using high-performance liquid chromatography (HPLC) (Kim H.-Y. et al., 2024), while the expression of inflammation-related genes was evaluated by quantitative polymerase chain reaction (qPCR) (Park et al., 2024). Protein-level changes in inflammatory mediators were also assessed using enzyme-linked immunosorbent assay (ELISA). Collectively, this study aimed to evaluate the anti-inflammatory potential of *S. anglica* and to provide insight into its relevance as a pharmacologically active natural resource.

## 2 Materials and methods

### 2.1 Salt marsh plants collection and preparation

*Sporobolus anglicus* (C.E.Hubb.) P.M.Peterson and Saarela [Poaceae, syn.: *S. anglica* C.E.Hubb.] was collected from coastal salt marshes in Ganghwa Island, Incheon, South Korea, during the peak growing season. After rinsing with distilled water, the plant material was dried in an oven at 40 °C for 24–72 h, ground into a fine powder, and stored at −20 °C until extraction. For initial screening, *S. anglica* specimens were obtained from the National Marine Biodiversity Institute of Korea (MABIK), where the species had already been taxonomically authenticated and catalogued as specimens collected from Ganghwa Island, Incheon, Republic of Korea. Fresh *S. anglica* specimens used in the present study were independently collected from Ganghwa Island (5 June 2018) and authenticated at Incheon National University, where voucher specimen preparation and species identification were performed. Detailed voucher specimen information is publicly available through the Blue Carbon Research Center of the Research Institute for Basic Sciences, Incheon National University (http://www.bluecarbon-ribs.re.kr/mng/bis/iubcBioSpeciesListView.do?pageIndex=1#1). Detailed information on plant collection, preprocessing, drying, and extraction procedures, together with extraction yield and batch-to-batch reproducibility, is summarized in Supplementary Table S1.

### 2.2 Extraction and phytochemical characterization

Powdered *S. anglica* (100 g) was extracted with 70% ethanol at room temperature for 48 h with intermittent mixing. The extract was filtered through Whatman No. 1 filter paper (GE Healthcare, Chicago, United States), concentrated under reduced pressure using a rotary evaporator (EYELA, Tokyo Rikakikai Co., Ltd., Tokyo, Japan) at 40 °C, lyophilized using a freeze-dryer (Operon, Seoul, Korea), and stored at −80 °C until use.

### 2.3 Cell culture and inflammatory stimulation

HaCaT human keratinocytes (ATCC, Manassas, United States) were maintained in Dulbecco’s Modified Eagle Medium (DMEM; Gibco, Grand Island, United States) supplemented with 10% fetal bovine serum (FBS; Gibco) and 1% penicillin–streptomycin (P/S; Gibco). Cells were incubated at 37 °C in a humidified atmosphere containing 5% CO_2_. For inflammatory stimulation, cells were seeded in 6-well plates at a density of 5 × 10^5 cells/well and treated with *S. anglica* extract (SAE) and tumor necrosis factor-alpha (TNF-α) and interferon-gamma (IFN-γ) (10 ng/mL each; BD Biosciences, San Jose, CA, United States) for 24 h.

### 2.4 Cell viability assay

Cell viability was evaluated using an MTT assay (Sigma-Aldrich, St. Louis, United States) as previously described (Mehta et al., 2024). Lyophilized SAE was dissolved in DMSO to prepare a stock solution. HaCaT cells were seeded in 96-well plates at a density of 1 × 10^5 cells/well and treated with *S. anglica* extract (0–500 μg/mL) for 24 h. MTT solution (5 mg/mL, 10 µL) was added to each well and incubated for 4 h at 37 °C. Formazan crystals were dissolved in dimethyl sulfoxide (DMSO), and absorbance was measured at 570 nm using a microplate spectrophotometer (Varioskan Lux, Thermo Fisher Scientific, Waltham, United States). Each treatment was performed in triplicate wells across three independent biological experiments (n = 3). Cell viability ≥70% relative to the untreated control was considered non-cytotoxic, in accordance with the ISO 10993–5 international standard for *in vitro* cytotoxicity evaluation.

### 2.5 Real-time quantitative polymerase chain reaction (RT-qPCR)

Total RNA was isolated using TRIzol reagent (Invitrogen) according to the manufacturer’s protocol. RNA purity and concentration were measured using a NanoDrop spectrophotometer (DaeMyung Science, Seoul, Korea), and RNA integrity was verified using an Agilent 2100 Bioanalyzer (Agilent Technologies, Waldbronn, Germany). cDNA was synthesized using a reverse transcription kit (Toyobo, Osaka, Japan). Quantitative real-time PCR was performed using SYBR Green Master Mix in a CFX Connect detection system (Bio-Rad Laboratories, Hercules, United States). Relative expression levels of TARC/CCL17 and MDC/CCL22 were calculated using the comparative Ct (ΔΔCt) method with GAPDH as the internal control. All reactions were performed in technical duplicate across three independent biological experiments (n = 3).

### 2.6 Library preparation and sequencing

For transcriptomic analysis, HaCaT cells were treated with SAE at 250 μg/mL, the highest non-cytotoxic concentration that also produced the greatest suppression of TARC and MDC expression. RNA sequencing libraries were prepared using the QuantSeq 3′mRNA-Seq Library Prep Kit (Lexogen, Vienna, Austria) following the manufacturer’s protocol. Sequencing was performed using single-end 75 bp reads on an Illumina NextSeq 500 platform (Illumina, Inc., San Diego, United States). QuantSeq 3′mRNA-Seq reads were aligned to the reference genome using Bowtie2 (Langmead and Salzberg, 2012). Differentially expressed genes (DEGs) were analyzed using edgeR within the Bioconductor R package, and read coverage was assessed using BEDtools (Quinlan and Hall, 2010). Functional classification was performed using DAVID and PubMed/MEDLINE resources (Gentleman et al., 2004). Raw read counts, normalized expression values, and fold changes for all genes are provided in Supplementary Table S2. RNA-seq was performed on one sample per condition (untreated control, TNF-α/IFN-γ, and TNF-α/IFN-γ + SAE 250 μg/mL). The RNA-seq data have been deposited in the NCBI GEO database under accession number GSE351832.

### 2.7 Nuclear and cytosolic protein extraction

For separation of cytosolic and nuclear proteins, cells were washed with cold PBS, collected by scraping, and processed using the Nuclear Extract Kit (Active Motif, Carlsbad, United States) according to the manufacturer’s instructions. The resulting cytosolic and nuclear fractions were stored at −80 °C until analysis. These fractions were used for the analysis of NF-κB p65 and IκB-α.

### 2.8 Western blot analysis

Cell lysates were prepared using RIPA buffer supplemented with protease and phosphatase inhibitors 1,2 (Sigma-Aldrich, St. Louis, United States). Protein concentration was determined using a BCA assay kit (Thermo Fisher Scientific). Equal amounts of protein (30 μg) were separated on SDS-PAGE gels, transferred to PVDF membranes (Bio-Rad Laboratories), blocked with 5% skim milk in TBST, and incubated overnight with primary antibodies against ERK1/2, phospho-ERK1/2, JNK1/2, phospho-JNK1/2, p38 MAPK, phospho-p38 MAPK, STAT1, phospho-STAT1, NF-κB p65, IκB-α, histone H1, α-tubulin, and GAPDH (1:1,000 dilution, Cell Signaling Technology, Beverly, MA, United States) (Gao et al., 2024). HRP-conjugated secondary antibodies (1:2,000 dilution) were applied for 1 h, and signals were visualized using an enhanced chemiluminescence (ECL) detection system (Bio-Rad). Band intensities were quantified by densitometry using Bio-Rad Image Lab software from three independent biological experiments (n = 3). Phosphorylated proteins were normalized to their corresponding total protein levels, whole-cell lysate targets were normalized to GAPDH, and nuclear and cytoplasmic fractions were normalized to histone H1 and α-tubulin, respectively.

### 2.9 Immunofluorescence staining (IF)

HaCaT cells were seeded on glass coverslips in 24-well plates and treated with *S. anglica* extract following TNF-α and IFN-γ stimulation. After 24 h, cells were fixed with 4% paraformaldehyde (Sigma-Aldrich) for 15 min and permeabilized with 0.1% Triton X-100 in PBS for 10 min. After blocking with 5% bovine serum albumin (Sigma-Aldrich) for 1 h, cells were incubated overnight at 4 °C with primary antibodies against NF-κB p65 (Cell Signaling Technology). Cells were then incubated with Alexa Fluor 488-conjugated anti-rabbit IgG secondary antibody (Cell Signaling Technology) for 1 h in the dark. Fluorescence images were acquired using a confocal microscope (Leica Microsystems, Wetzlar, Germany) and analyzed with ImageJ software.

### 2.10 Enzyme-linked immunosorbent assay (ELISA)

Culture supernatants were collected after extract treatment and analyzed for TARC/CCL17 and MDC/CCL22 using ELISA kits (R&D Systems, Minneapolis, MN, United States). Absorbance values were recorded at 450 nm using a microplate reader (Varioskan Lux, Thermo Fisher Scientific). Each condition was assayed in duplicate across three independent biological experiments (n = 3).

### 2.11 High-performance liquid chromatography (HPLC)

HPLC analysis was performed using an Agilent 1200 series system (Agilent Technologies, Waldbronn, Germany) equipped with a photodiode array (PDA) detector. The mobile phase consisted of acetonitrile and 0.1% formic acid in gradient elution mode. The column temperature was maintained at 30 °C, and the flow rate was 1.0 mL/min. Peaks were identified by comparison of retention times and spectral data with reference standards.

### 2.12 Statistical analysis

Statistical significance for Western blot data was assessed using one-way analysis of variance (ANOVA) followed by Tukey’s multiple comparisons test using SigmaStat software. For all other data, statistical significance was assessed using one-way ANOVA followed by Dunnett’s multiple comparisons test using GraphPad Prism 9.0 software. All quantitative experiments were performed using three independent biological replicates (n = 3), and data are presented as the mean ± standard deviation (SD). Statistical significance is indicated in the figures as follows: #P < 0.05, ##P < 0.01, ###P < 0.001, ####P < 0.0001 versus the untreated control group; *P < 0.05, **P < 0.01, ***P < 0.001, ****P < 0.0001 versus the TNF-α/IFN-γ-treated group.

## 3 Results

### 3.1 SAE effectively suppresses pro-inflammatory chemokine expression without inducing cytotoxicity in IFN-γ- and TNF-α-stimulated HaCaT cells

To evaluate the anti-inflammatory potential of SAE derived from *S. anglica*, its effects on cell viability and chemokine expression were investigated in TNF-α and IFN-γ stimulated HaCaT cells (Figure 1). The expression levels of TARC/CCL17 and MDC/CCL22 were analyzed at both the protein and mRNA levels.

- The cytotoxic effects of SAE were first assessed using an MTT assay. HaCaT cells were treated with increasing concentrations of SAE (5–500 μg/mL) for 24 h. A substantial reduction in cell viability was observed at 500 μg/mL, where viability decreased to approximately 30%, indicating cytotoxic effects at this concentration. In contrast, treatment with concentrations up to 250 μg/mL maintained cell viability at approximately 85%, suggesting that SAE does not exert significant cytotoxicity within this range. Based on these findings, 250 μg/mL was defined as the upper limit for non-cytotoxic conditions and was used for subsequent analyses.
- To determine whether SAE modulates inflammatory chemokine secretion, HaCaT cells were stimulated with TNF-α and IFN-γ to induce an inflammatory response. As expected, cytokine stimulation markedly increased the secretion of MDC and TARC, reaching approximately 200 and 100 pg/mL, respectively. Co-treatment with SAE resulted in a progressive decrease in chemokine levels in a concentration-dependent manner. Notably, treatment with 250 μg/mL SAE led to a pronounced reduction in both MDC and TARC levels compared to the cytokine-stimulated group, indicating that SAE effectively suppresses inflammatory chemokine secretion at the protein level.
- To further investigate whether this suppression occurs at the transcriptional level, the mRNA expression of TARC/CCL17 and MDC/CCL22 was analyzed by RT-qPCR under the same experimental conditions. Stimulation with TNF-α and IFN-γ significantly increased MDC expression by approximately 8-fold and TARC expression by approximately 1.5-fold relative to untreated controls. Treatment with SAE significantly attenuated these increases in a concentration-dependent manner. At 250 μg/mL, MDC expression was reduced by more than 70% compared to the cytokine-stimulated group. In addition, TARC expression was suppressed to levels comparable to or lower than those observed in the untreated control group. These findings indicate that SAE suppresses TARC/CCL17 and MDC/CCL22 at the transcriptional level; however, the concentration-response patterns did not fully parallel the corresponding protein-level changes, particularly for TARC, which may reflect differences in the kinetics of mRNA turnover versus protein secretion.

**FIGURE 1:**
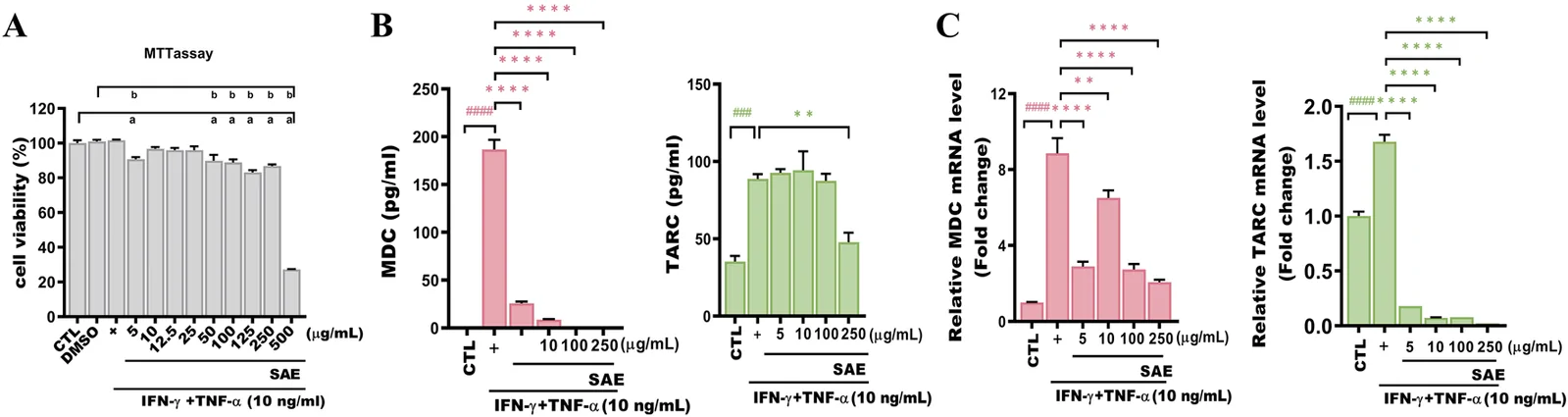
**(A)** Cell viability was analyzed by MTT assay. HaCaT cells were seeded into 96-well plates and treated with SAE at 5–500 μg/mL for 24 h. Statistical comparisons were performed between control vs. each SAE concentration (denoted as *a*) and between DMSO vs. each SAE concentration (denoted as *b*); a: *p* < 0.05, b: *p* < 0.05. **(B)** Inflammatory conditions were induced by treating HaCaT cells with IFN-γ and TNF-α (10 ng/mL each) with SAE at concentrations of 5, 10, 100, and 250 μg/mL for 24 h. After incubation, culture supernatants were collected, and the levels of secreted TARC/CCL17 and MDC/CCL22 were measured using ELISA. **(C)** In parallel, total RNA was extracted from identically treated cells to quantify mRNA levels of TARC/CCL17 and MDC/CCL22 using real-time qPCR. Each result reflects the mean ± SD obtained from three distinct experimental trials. Statistical significance was assessed by one-way ANOVA followed by Dunnett’s multiple comparisons test. Statistical significance was indicated as follows: **p < 0.01, ****p < 0.0001 versus TNF-α/IFN-γ-stimulated group; ###p < 0.001, ####p < 0.0001 versus control group.

Taken together, these results indicate that SAE attenuates inflammatory chemokine production in cytokine-stimulated keratinocytes without inducing cytotoxicity at effective concentrations.

### 3.2 SAE attenuates cytokine-induced activation of MAPK and STAT1 signaling pathways in a dose-dependent manner in HaCaT keratinocytes

To further elucidate the molecular mechanisms underlying the anti-inflammatory effects of SAE, its impact on key intracellular signaling pathways was examined in HaCaT cells stimulated with TNF-α and IFN-γ (10 ng/mL each) (Figure 2).

**FIGURE 2:**
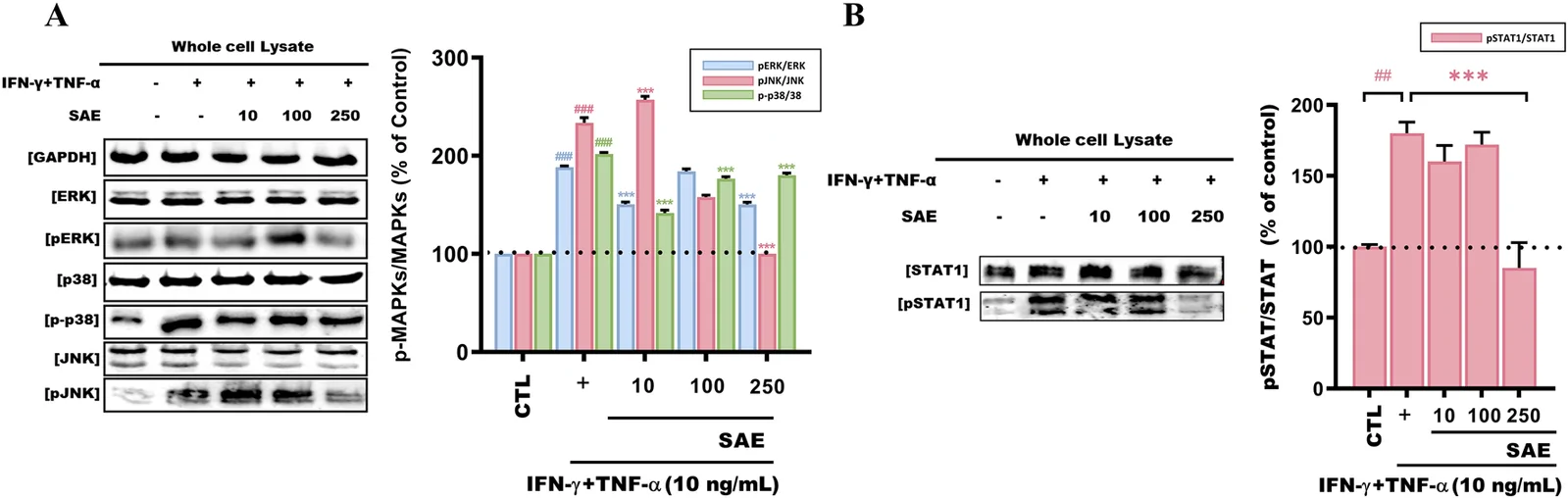
HaCaT cells were incubated with different concentrations of SAE with TNF-α and IFN-γ (10 ng/mL each) for 24 h to induce an inflammatory response. **(A)** Phosphorylated and total ERK, JNK, and p38 were detected by Western blot analysis, with GAPDH used as a loading control, and the band intensities of the phosphorylated proteins were quantified relative to their total protein counterparts. *(B)* Phosphorylated and total STAT1 were detected by Western blot analysis, and the pSTAT1/STAT1 ratio was quantified. Data are presented as the mean ± standard deviation (SD) from three independent experiments (n = 3). Statistical significance was assessed by one-way ANOVA followed by Tukey’s multiple comparisons test. Statistical significance was indicated as follows: #: comparison versus untreated control (##*p* < 0.01, ###*p* < 0.001, ####*p* < 0.0001); *: comparison versus TNF-α/IFN-γ-stimulated group (**p < 0.01, ***p < 0.001, ****p < 0.0001).

(a) Exposure to pro-inflammatory cytokines led to a marked increase in the phosphorylation of MAPK pathway components, including ERK, JNK, and p38, confirming activation of these signaling cascades in response to inflammatory stimulation. Treatment with SAE resulted in a concentration-dependent attenuation of MAPK activation. Phosphorylation levels of JNK were substantially reduced at higher concentrations of SAE, whereas ERK and p38 phosphorylation showed a comparatively moderate decrease. Despite these changes in phosphorylation status, the total protein levels of ERK, JNK, and p38 remained largely unchanged across all experimental groups. This indicates that SAE primarily modulates kinase activation rather than affecting protein expression levels. (b) The regulatory effect of SAE on the STAT1 signaling pathway was also assessed under the same experimental conditions. Cytokine stimulation significantly elevated p-STAT1 levels, indicating activation of STAT1-mediated signaling. In contrast, SAE treatment led to a progressive decrease in p-STAT1 levels in a dose-dependent manner. Importantly, total STAT1 protein expression did not exhibit notable changes, resulting in a marked reduction in the p-STAT1/STAT1 ratio following SAE treatment. Taken together, these results demonstrate that SAE effectively attenuates cytokine-induced activation of MAPK and STAT1 signaling pathways in HaCaT cells, predominantly through inhibition of phosphorylation events. The simultaneous modulation of multiple inflammatory signaling pathways suggests a coordinated regulatory effect of SAE at the post-translational level.

### 3.3 SAE suppresses TNF-α/IFN-γ-induced activation of the NF-κB signaling pathway in HaCaT keratinocytes

The effect of SAE on NF-κB signaling was examined by evaluating p65 nuclear translocation and cytoplasmic IκB-α levels in HaCaT cells following stimulation with TNF-α and IFN-γ (10 ng/mL each) (Figure 3). Cells were treated with SAE (250 μg/mL) and TNF-α and IFN-γ (10 ng/mL each) for 24 h. Western blot analysis revealed that cytokine stimulation markedly increased nuclear accumulation of NF-κB p65, accompanied by a reduction in cytoplasmic IκB-α levels, consistent with activation of the NF-κB signaling pathway. In contrast, SAE treatment attenuated the nuclear localization of p65 and preserved IκB-α expression in the cytoplasmic fraction. For accurate comparison between subcellular compartments, nuclear and cytoplasmic proteins were normalized to histone H1 and α-tubulin, respectively. These observations were further supported by immunofluorescence staining. DAPI staining was used to visualize nuclei, while NF-κB p65 was detected using a fluorescently labeled antibody. In cytokine-treated cells, p65 localization was predominantly observed in the nucleus, indicating activation of NF-κB signaling. In contrast, SAE-treated cells displayed reduced nuclear p65 signals, with a more diffuse distribution in the cytoplasm. Taken together, these results demonstrate that SAE inhibits cytokine-induced NF-κB activation by preventing p65 nuclear translocation and maintaining cytoplasmic IκB-α levels.

**FIGURE 3:**
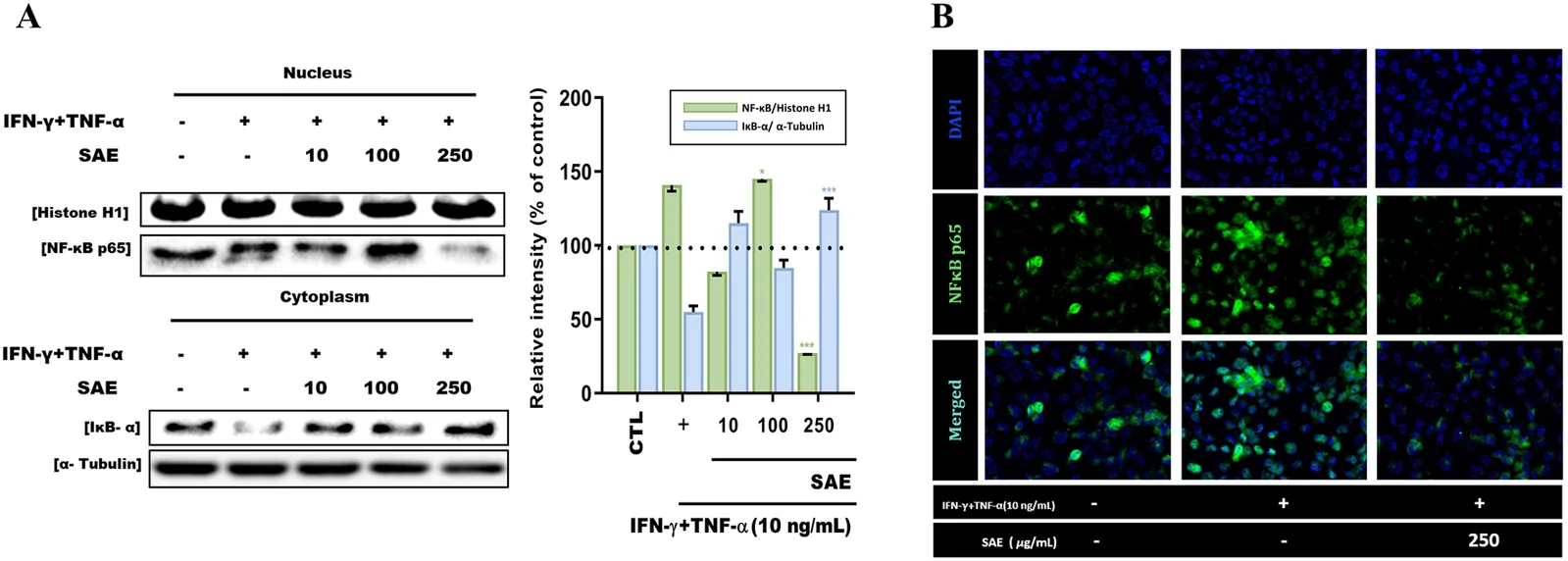
HaCaT cells were treated with SAE (10, 100, and 250 μg/mL for Western blotting; 250 μg/mL for immunofluorescence) and TNF-α and IFN-γ (10 ng/mL each) for 24 h. **(A)** Western blotting was carried out to detect NF-κB p65 in nuclear fractions and IκB-α in cytoplasmic fractions. Histone H1 and α-tubulin served as loading controls for nuclear and cytoplasmic proteins, respectively. **(B)** For immunofluorescence analysis, NF-κB p65 was detected using a rabbit-derived primary antibody, followed by incubation with an Alexa Fluor 488-labeled goat anti-rabbit IgG. Nuclear counterstaining was done with DAPI in mounting medium. Protein band intensities were measured via densitometric analysis. Data are shown as mean ± standard deviation (SD), based on three biologically independent experiments (n = 3). Statistical significance was assessed by one-way ANOVA followed by Tukey’s multiple comparisons test. Statistical significance was indicated as follows: *p < 0.05, ***p < 0.001 versus TNF-α/IFN-γ-stimulated group.

### 3.4 HPLC-based identification of putative anti-inflammatory metabolites in the ethanol extract of SAE

The chemical composition of SAE was analyzed by high-performance liquid chromatography (HPLC) to identify putative bioactive metabolites (Figure 4). The chromatographic profile of the ethanol extract revealed multiple peaks, indicating the presence of diverse metabolites. Notably, a prominent peak detected at a retention time of 17.091 min closely matched that of the iso-orientin standard analyzed under identical conditions, suggesting the presence of iso-orientin or a structurally related flavonoid in the extract. To estimate its abundance, peak areas were compared with those of the iso-orientin standard. The corresponding peak in the SAE exhibited approximately 50%–60% of the standard peak area, yielding an estimated concentration of ∼0.8–1.0 μg/mL. These findings indicate that iso-orientin-like metabolites are present as relatively abundant levels in SAE and may contribute to its observed bioactivity.

**FIGURE 4:**
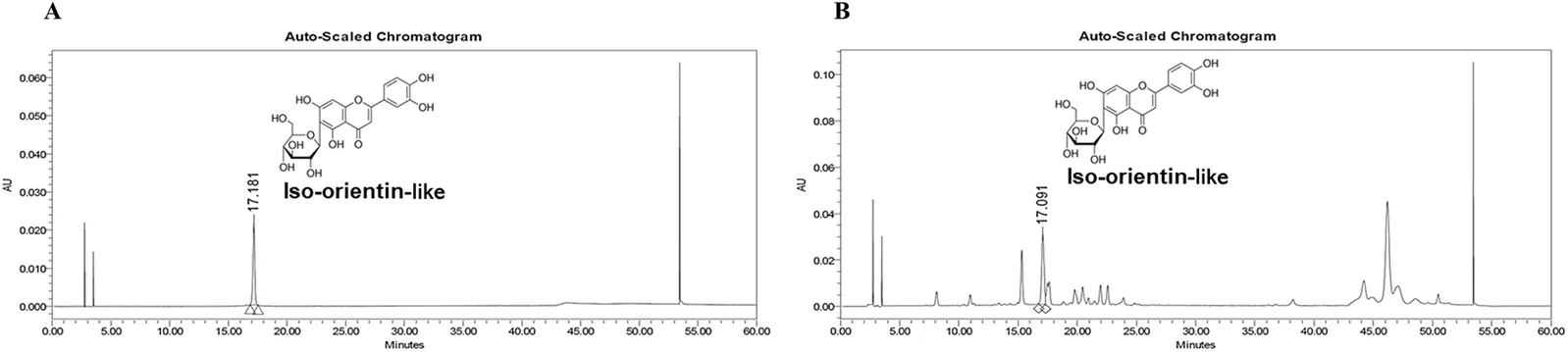
Identification of iso-orientin-like metabolites in SAE by HPLC analysis. **(A)** Chromatographic profile of the iso-orientin standard. **(B)** Chromatogram of the SAE, displaying a peak at 17.091 min corresponding to the retention time of the iso-orientin standard. HPLC analysis was performed using an Agilent 1200 system with a mobile phase consisting of acetonitrile and 0.1% formic acid in gradient elution mode. The injection volume was 20 μL, and the C18 column (4.6 × 250 mm, 5 μm) was used.

### 3.5 RNA-seq analysis reveals broad transcriptional reprogramming of inflammatory and immune-related pathways following SAE treatment

To further explore molecular mechanisms beyond the canonical NF-κB, STAT1, and MAPK pathways identified above, transcriptomic profiling was performed to investigate whether SAE modulates broader inflammatory and immune-associated signaling networks (Gibbs et al., 2025).

Differential expression analysis identified a substantial number of genes that were oppositely regulated between cytokine stimulation and SAE treatment. Among these, 672 genes exhibited contra-regulated expression patterns, indicating that SAE effectively reversed a large proportion of TNF-α/IFN-γ-induced transcriptional changes (Figure 5A). Consistent with this observation, volcano plot analysis demonstrated that SAE upregulated anti-inflammatory and regulatory genes, including HMOX1, PTPN6, PTGR1, TGFBR3, and NR4A2, while suppressing multiple inflammation-associated genes, including TARC/CCL17, MDC/CCL22, CXCL9, IL1B, and CXCL2.

**FIGURE 5:**
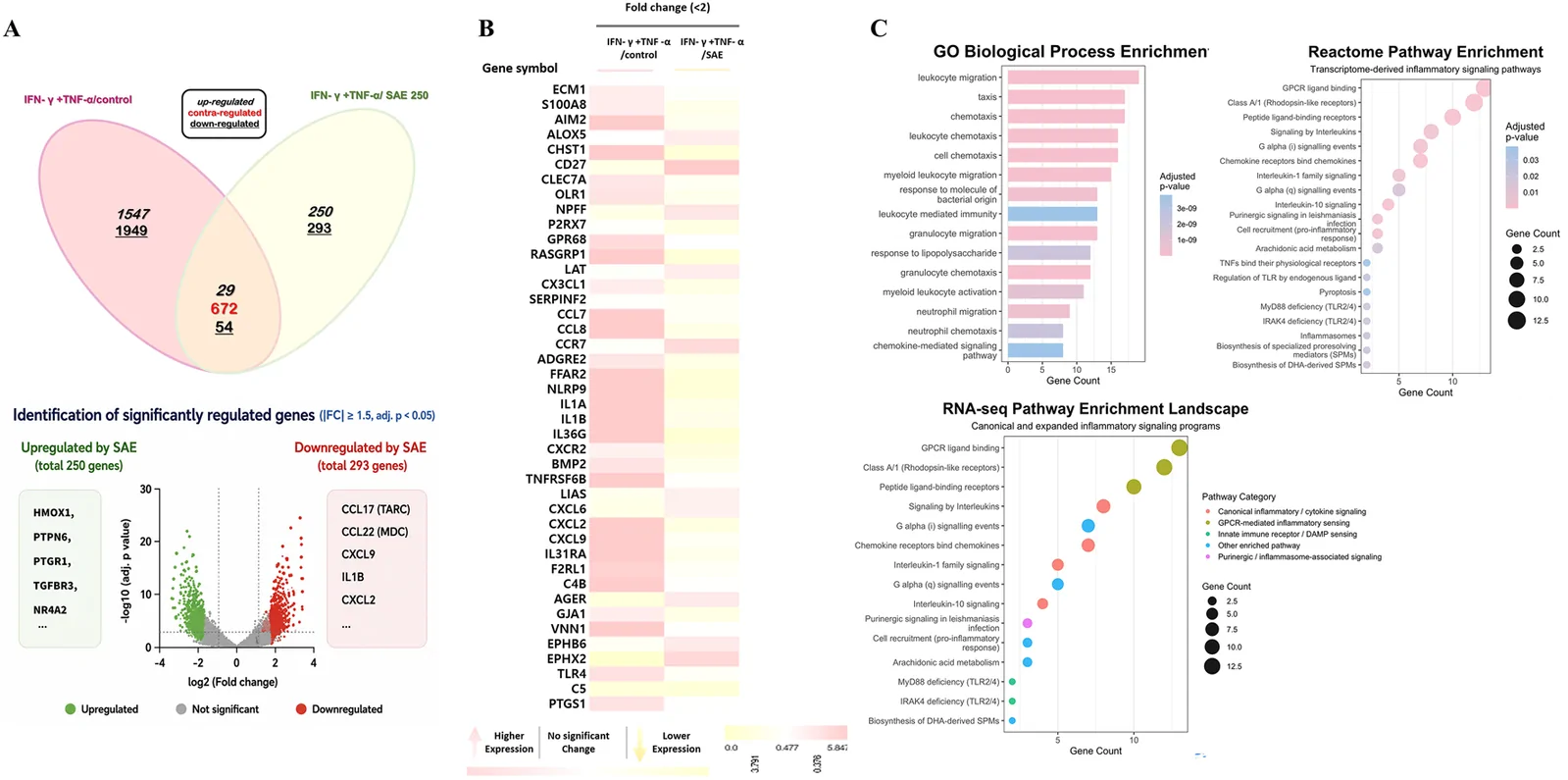
**(A)** Identification of differentially expressed genes (DEGs) in TNF-α/IFN-γ-stimulated cells and SAE-treated cells. The Venn diagram shows the overlap of significantly regulated genes between cytokine stimulation and SAE treatment. Italicized numbers indicate upregulated genes, underlined numbers indicate downregulated genes, and red numbers represent contra-regulated genes (|FC| ≥ 1.5, adjusted p < 0.05). The volcano plot highlights representative genes upregulated or downregulated by SAE treatment. **(B)** Heatmap visualization of representative contra-regulated inflammatory genes. Normalized expression values illustrate transcriptional changes induced by TNF-α/IFN-γ stimulation and their modulation by SAE treatment. Genes shown in the heatmap were selected using a ≥2-fold/≤0.5-fold change (P ≤ 0.05) criterion, distinct from the overall DEG criterion in **(A)**. **(C)** Functional enrichment analysis of contra-regulated genes using GO Biological Process and Reactome pathway databases. Enrichment analyses reveal biological processes associated with leukocyte migration, chemotaxis, cytokine signaling, innate immune responses, GPCR-mediated inflammatory signaling, and inflammatory cell recruitment. Bubble size indicates gene count, and color represents adjusted p-value.

To further characterize the biological relevance of these transcriptomic alterations, representative inflammation-related genes were visualized using heatmap analysis (Figure 5B). TNF-α/IFN-γ stimulation markedly increased the expression of genes associated with inflammatory signaling, leukocyte recruitment, chemotaxis, innate immune activation, and cytokine responses. SAE treatment substantially attenuated the expression of these genes, including members of the CXCL and CCL chemokine families, IL1A, IL1B, IL36G, CXCL2, CXCL9, and TNFRSF6B, restoring expression profiles toward a less inflammatory state.

To gain mechanistic insight into the biological processes affected by SAE, Gene Ontology (GO) and pathway enrichment analyses were performed using contra-regulated genes (Figure 5C). GO enrichment analysis revealed significant overrepresentation of processes associated with leukocyte migration, chemotaxis, granulocyte migration, neutrophil chemotaxis, and chemokine-mediated signaling pathways. Consistent with these findings, Reactome and integrated pathway landscape analyses identified multiple inflammation-associated pathways, including interleukin signaling, chemokine receptor interactions, GPCR-mediated inflammatory signaling, innate immune receptor pathways, and purinergic signaling networks. Notably, several pathways linked to inflammatory cell recruitment and amplification of immune responses were among the most significantly enriched categories.

Collectively, these findings suggest that the anti-inflammatory activity of SAE extends beyond suppression of the canonical NF-κB, STAT1, and MAPK pathways. Transcriptomic profiling revealed coordinated regulation of genes involved in leukocyte recruitment, chemokine signaling, cytokine communication, and GPCR-associated inflammatory networks, providing broader insight into the molecular mechanisms underlying the anti-inflammatory effects of SAE.

### 3.6 Effect of SAE on NF-κB translocation and TARC/MDC production via the MAPK/STAT1 pathway, and putative contribution of iso-orientin-like metabolites

Figure 6 provides a schematic illustration summarizing the proposed mechanism of SAE-mediated anti-inflammatory effects. Based on the experimental findings, SAE inhibits the activation of MAPK (ERK, JNK, p38) and STAT1 signaling pathways in response to TNF-α and IFN-γ stimulation, accompanied by suppression of NF-κB nuclear translocation and reduced expression of TARC/CCL17 and MDC/CCL22. The causal sequence among these events was not directly tested in this study. A major peak observed in the HPLC analysis of SAE corresponded to the retention time of the iso-orientin standard under identical conditions, suggesting the presence of iso-orientin or a structurally related flavonoid. Based on its reported biological activities, iso-orientin-like metabolites may contribute to the observed anti-inflammatory effects of SAE.

**FIGURE 6:**
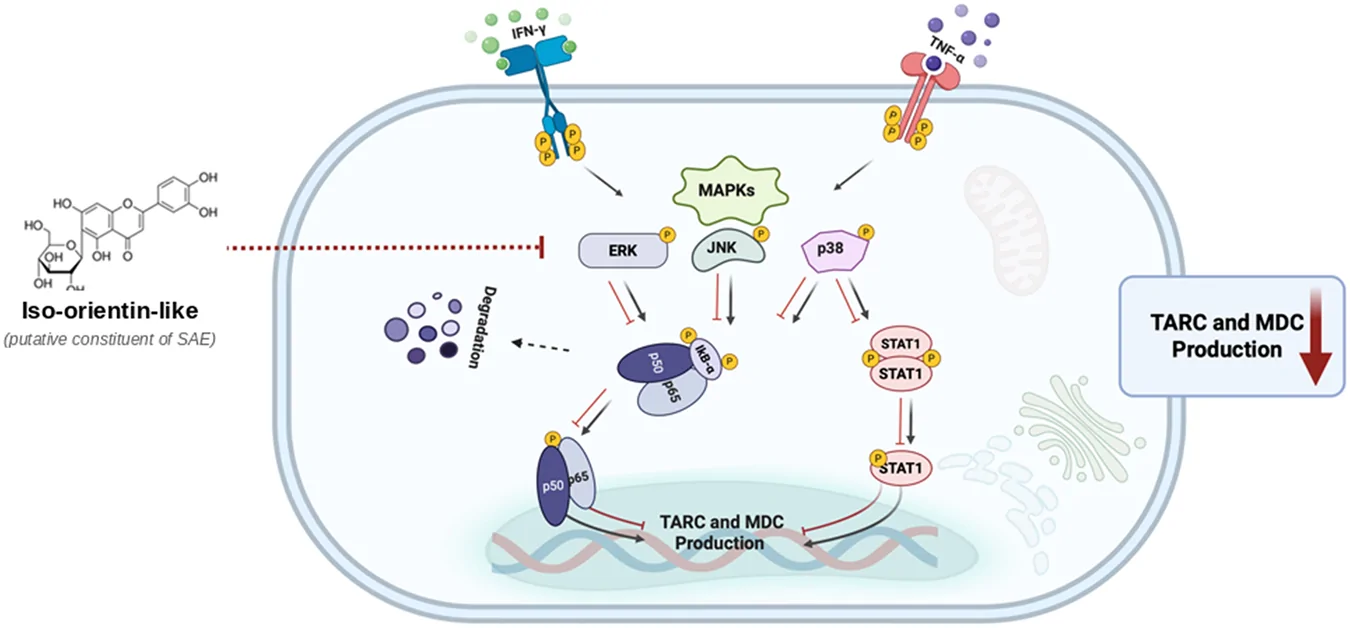
Visual summary of the inhibitory effect of SAE on nuclear NF-κB translocation and TARC/CCL17 and MDC/CCL22 production via the MAPK/STAT1 pathway, highlighting iso-orientin-like metabolites as a candidate bioactive metabolite.

## 4 Discussion

In the present study, we investigated the anti-inflammatory effects of *S. anglica* extract (SAE) using an *in vitro* keratinocyte-based model. We focused on the regulation of major pro-inflammatory chemokines and the signaling pathways associated with their expression. SAE significantly reduced the expression of TARC/CCL17 and MDC/CCL22 at both the mRNA and protein levels without inducing cytotoxicity within the tested concentration range. These findings indicate that SAE exerts anti-inflammatory activity under inflammatory conditions in keratinocytes. In addition, HPLC analysis revealed a major peak corresponding to the retention time of iso-orientin under the same analytical conditions, suggesting that iso-orientin or a structurally related metabolite may be present in SAE and may contribute to its biological activity.

Given the established roles of TARC/CCL17 and MDC/CCL22 in chronic inflammatory diseases such as atopic dermatitis (AD), their suppression is particularly relevant. These chemokines mediate the recruitment and activation of Th2 lymphocytes and eosinophils, which are central to AD pathogenesis (Horikawa et al., 2002). The observed inhibition of TARC and MDC by SAE suggests a potential role in modulating keratinocyte-driven inflammatory responses. This effect is consistent with previous reports showing that marine-derived bioactive metabolites, such as fucoxanthin, can regulate inflammatory mediator production and exert immunomodulatory effects (Kim et al., 2010).

The anti-inflammatory effects of SAE were concentration-dependent, with the most pronounced reduction in TARC and MDC levels observed at 250 μg/mL. This concentration was confirmed to be non-cytotoxic by MTT analysis, supporting its suitability for functional evaluation. The consistent suppression of chemokine expression at both mRNA and protein levels indicates that SAE regulates inflammatory responses at multiple stages of gene expression. These findings are consistent with previous studies demonstrating that marine-derived bioactive metabolites, such as fucoxanthin and phlorotannins, exert anti-inflammatory effects through the regulation of cytokine production.

To elucidate the molecular mechanisms underlying the anti-inflammatory effects of SAE, key signaling pathways associated with inflammatory responses were examined. SAE treatment markedly attenuated cytokine-induced phosphorylation of MAPK components (ERK, JNK, and p38) as well as STAT1 in TNF-α/IFN-γ-stimulated HaCaT cells. The inhibitory effects were most evident for JNK, while ERK and p38 phosphorylation showed a comparatively moderate reduction. These findings are consistent with previous reports indicating that marine-derived natural metabolites can suppress inflammatory responses through modulation of MAPK and STAT1 signaling pathways (Cheung et al., 2016). In addition to MAPK and STAT1, SAE also inhibited NF-κB signaling, as evidenced by the stabilization of IκB-α and reduced nuclear translocation of NF-κB p65. Taken together, these results suggest that SAE exerts its anti-inflammatory effects by coordinately targeting multiple signaling pathways, including MAPK, STAT1, and NF-κB, which are central regulators of inflammatory responses. This is mechanistically consistent with the RNA-seq results, which showed concurrent upregulation of negative regulators of these pathways, including PTPN6 (a STAT1 inhibitor) and NR4A2 (an NF-κB suppressor), together with downregulation of amplifying chemokines such as CXCL9 and CXCL2.

The detection of a peak corresponding to iso-orientin in the SAE suggests that this flavonoid may contribute to the observed anti-inflammatory effects. In the present study, the retention time of a major peak in the SAE chromatogram was consistent with that of the iso-orientin standard, and its estimated concentration was approximately 0.8–1.0 μg/mL. Although these findings suggest a potential role for iso-orientin in mediating the anti-inflammatory activity of SAE, its direct contribution remains to be further validated through metabolites-specific functional assays.

The present study demonstrated the anti-inflammatory activity of SAE in TNF-α/IFN-γ-stimulated keratinocytes and identified a chromatographic peak corresponding to the retention time of iso-orientin, suggesting the presence of iso-orientin-like metabolites in SAE. Previous studies have reported antioxidant, tyrosinase-inhibitory, pancreatic lipase-inhibitory, and anti-inflammatory activities of *S. anglica*, with its anti-inflammatory effects primarily demonstrated in macrophage-based inflammatory models (Kim et al., 2021; Song and Lee, 2022). Collectively, these findings extend the current pharmacological evidence for *S. anglica* by demonstrating its anti-inflammatory activity in a keratinocyte-based inflammatory model and suggesting the presence of a previously unreported iso-orientin-like metabolite in this species. Given that iso-orientin has been reported to exert anti-inflammatory effects in various experimental models (PMID: 28243356), its detection in SAE suggests that it may represent one of the bioactive metabolites contributing to the anti-inflammatory activity observed in the present study.

Beyond its pharmacological significance, the present findings also highlight the potential value of *S. anglica* as a sustainable marine bioresource. As an invasive halophyte that requires continuous removal along the Korean coastline, *S. anglica* generates substantial biomass that is largely discarded despite the associated management and disposal costs. The identification of pharmacologically active metabolites from this underutilized biomass supports a value-added approach to invasive biomass management and may contribute to the development of high-value marine-derived cosmetic and pharmaceutical resources.

Because all biological experiments in the present study were performed using the crude extract, the contribution of individual metabolites could not be distinguished, and the observed anti-inflammatory activity should therefore be interpreted as the biological activity of SAE as a whole rather than of any single metabolite. Future studies employing preparative liquid chromatography to isolate the fraction corresponding to the iso-orientin peak, followed by biological evaluation and structural confirmation using LC-MS/MS, will help clarify the contribution of iso-orientin to the biological activity of SAE. Such investigations will further strengthen the chemical characterization of SAE, facilitate the identification of its principal bioactive metabolites, and support the development of *S. anglica* as a sustainable marine-derived bioresource.

## Data Availability

The original contributions presented in the study are publicly available. This data can be found here: NCBI Gene Expression Omnibus (GEO), accession number GSE351832 (https://www.ncbi.nlm.nih.gov/geo/query/acc.cgi?acc=GSE351832).
