# Supplementary material for: Spartina anglica extract attenuates TNF-α/IFN-γ-induced inflammatory responses in HaCaT keratinocytes through suppression of the MAPK, NF-κB, and STAT1 signaling pathways
Source: Front Pharmacol. 2026 Oct 30;17:1931934. doi: 10.3389/fphar.2026.1931934 (PMC13759837; doi:10.3389/fphar.2026.1931934)
Supplement: Supplementary file 1 [file Table1.docx]

**Supplementary Table**

**Supplementary Table S1. Plant collection, authentication, and extraction information for *Spartina anglica* extract (SAE).**

| **Item** | **Description** |
| --- | --- |
| Plant material | *Spartina anglica* collected from Ganghwa Island, Incheon, Republic of Korea (June 5, 2018) |
| Fresh plant weight | 10 kg |
| Washing | Thoroughly washed with tap water to remove salt and debris |
| Drying | 40°C for 24-72 h |
| Dried powder obtained | 652.15 g |
| Amount used per extraction | 100 g dried powder |
| Extraction solvent | 70% (v/v) ethanol |
| Solvent-to-solid ratio | 1:10 (w/v) |
| Extraction conditions | Room temperature, 48 h |
| Filtration | Whatman filter paper |
| Concentration | Rotary evaporator at 45°C (Rotary evaporator N-1000, EYELA, Tokyo Rikakikai Co., Ltd., Tokyo, Japan) |
| Extraction yield | 10.8% (w/w) |
| Batch reproducibility | Independent extractions performed using the same protocol yielded consistent extraction yields of approximately 8-10.8% (w/w) |
| Extraction protocol disclosure | Korean Patent KR20220059060A |
